# Recombinant Viruses and Early Global HIV-1 Epidemic

**DOI:** 10.3201/eid1007.030904

**Published:** 2004-07

**Authors:** Marcia L. Kalish, Kenneth E. Robbins, Danuta Pieniazek, Amanda Schaefer, Nzila Nzilambi, Thomas C. Quinn, Michael E. St. Louis, Ae S. Youngpairoj, Jonathan Phillips, Harold W. Jaffe, Thomas M. Folks

**Affiliations:** * Centers for Disease Control and Prevention, Atlanta, Georgia, USA;; † Project SIDA, Kinshasa, Zaire;; ‡ National Institutes of Health, Bethesda, Maryland, USA

**Keywords:** HIV-1 infections, Africa, Central/ep [epidemiology], HIV-1/cl [classification], Kinshasa, Zaire, molecular sequence data, recombination, genetic, variation, genetics, phylogeny, genes, env/ge [genetics], genes, gag/ge [genetics], research

## Abstract

Central Africa was the epicenter of the HIV type 1 (HIV-1) pandemic. Understanding the early epidemic in the Democratic Republic of the Congo, formerly Zaire, could provide insight into how HIV evolved and assist vaccine design and intervention efforts. Using enzyme immunosorbent assays, we tested 3,988 serum samples collected in Kinshasa in the mid-1980s and confirmed seroreactivity by Western blot. Polymerase chain reaction of *gag* p17, *en*v C2V3C3, and/or gp41; DNA sequencing; and genetic analyses were performed. Gene regions representing all the HIV-1 group M clades and unclassifiable sequences were found. From two or three short gene regions, 37% of the strains represented recombinant viruses, multiple infections, or both, which suggests that if whole genome sequences were available, most of these strains would have mosaic genomes. We propose that the HIV epidemic was well established in central Africa by the early 1980s and that some recombinant viruses most likely seeded the early global epidemic.

## Abstract 2

HIV strains from Zaire indicate the HIV epidemic in Kinshasa was mature by the mid-1980s.

Almost 70% of all HIV type 1 infections worldwide are found in sub-Saharan Africa, and central Africa is the only region where all HIV-1 groups and group M subtypes have been identified (*1**–**3*). This broad range of diversity suggested central Africa as the epicenter of the HIV-1 pandemic (*1*). Phylogenetic reconstruction has shown that HIV-1 appears in three distinct lineages, groups M, N, and O (*4**–**7*), and each is believed to have arisen through separate zoonotic infections with chimpanzee simian immunodeficiency virus strains in central Africa (*8**,**9*). HIV-1 group M viruses are primarily responsible for the current global epidemic, while group O infections are far fewer and generally found in west-central Africa. Rare strains of group N viruses have been identified in Cameroon, also located in western equatorial Africa. The group M viruses can be subdivided into 9 subtypes, A–D, F–H, J, and K, and at least 14 circulating recombinant forms (CRFs) (http://hiv-web.lanl.gov). These phylogenetic clusterings of HIV strains, subtypes, and CRFs are important records of the epidemic histories of HIV, and they highlight the differences in their geographic distributions and areas where they are endemic.

Some of the earliest characterized whole-genome HIV-1 sequences were collected in Zaire, currently called the Democratic Republic of the Congo (DRC), in the late 1970s and early 1980s. During this time, Kinshasa, the capital of Zaire, was the largest urban area in central Africa, with a population estimated at 2.5 million (*10*). From 1984 through 1991, the Zaire Department of Public Health conducted a long-term collaborative HIV research and surveillance program, Project SIDA, with the U.S. Department of Health and Human Services and the Institute of Tropical Medicine, Antwerp, Belgium. In 1991, Project SIDA was abruptly terminated because of civil unrest.

A low and stable HIV prevalence in Zaire/DRC from 1976–1997 (*11**,**12*) has been documented, despite social and political upheavals. A genetic survey of HIV strains performed in three regions of the DRC in 1997 demonstrated a high diversity of HIV-1 group M subtypes (*1*). Serum samples, available from Project SIDA, offered us the opportunity to characterize HIV strains collected in Kinshasa, Zaire, in 1984 and 1986 from employees at Mama Yemo Hospital (*13**,**14*). The results provide new insights into the dynamics of HIV infections in a low-prevalence area where multiple subtypes cocirculate, early in the global HIV-1 epidemic.

## Methods

### Specimens and Serologic Analyses

Serum samples acquired through Project SIDA from a 1984 and 1986 cross-sectional study of HIV infection among hospital employees at Mama Yemo Hospital in Kinshasa, Zaire were sent to the National Institutes of Health (NIH) and stored at –20°C. A total of 3,988 serum samples were sent to the Centers for Disease Control and Prevention for serologic and genetic analysis. Specimens numbered 30,000 through 32,000 were collected in 1984. Samples numbered 33,000 through 35,585 were collected among the same population in 1986. The specimens tested represent a convenience sampling from the U.S. repository at NIH and did not include all or only the samples in the original study. The volumes of the specimens ranged from approximately 200 µL to 4 mL. All samples were tested in two separate whole viral-lysate enzyme immunosorbent assay (EIA) for antibodies to HIV-1 and HIV-2 (Genetic Systems, Redmond, WA). Samples with insufficient volumes of serum were excluded from the analysis. Serum specimens reactive by EIA were further tested by using an HIV-1/2 Western blot assay (Genelabs Diagnostics, Singapore, version 2.2).

### RNA Extraction, RT, and PCR

Samples with sufficient volumes of serum for reverse transcription–polymerase chain reaction (RT-PCR) amplification with three different sets of primers were selected for further study. RNA was extracted from serum by either the NucliSens nucleic acid manual or automated protocols (Organon Teknika, Boxtel, the Netherlands). RT-PCR was performed by using the Promega RT kit (Promega, Madison, WI) following the manufacturer's protocol. PCR reactions were placed in a GeneAMP 9600 thermocycler (Perkin-Elmer Cetus, Norwalk, CT) for 35–40 cycles as follows: 94°C for 30s, 55°C for 30s, and 72°C for 60s for all but *env* gp41, which was at 50°C for 30s and 72°C for 60s, with the final extension at 72°C for 5 min. Nested PCR products were electrophoresed in 1.5% agarose gels (Gibco, Grand Island, NY) and visualized with ethidium bromide staining. To avoid contamination, sample processing and pre-PCR set-up were performed in different rooms than post-PCR manipulations. All samples with discordant phylogenies in different gene regions were verified by having a different person reamplify and resequence the gene regions from a second, unopened vial of serum, if available, or from the previously used vial.

### Primers

For nested PCR amplification of 380-bp *env* C2V3C3 fragments, two sets were used: JH44F and JH35MR (outside) and JH33F and JH48R (nested) (*15*), and MK369 and MK616 (outside) and MK650 and CO601 (nested) (*15*). Primers for PCR amplification of a 475-bp *gag* p17 fragment were CL1028 and AB1033 (outside) and CL1029 and AB1032 (nested) (*16*); those used for a 460-bp fragment of *env* gp41 were GP40F1 and GP41R1 (outside) and GP46F2 and GP48R2 or GP47R2 (nested) (*17*).

### Sequencing PCR Products

The PCR-amplified products were purified with Qiagen PCR purification kits (Qiagen Inc, Chatsworth, CA) and directly sequenced by using the ABI PRISM Big Dye Terminator Cycle Sequencing Ready Reaction Kit (Applied Biosystems/Perkin Elmer, Foster City, CA) and both forward and reverse nested PCR primers. The sequencing reactions were purified by using the DyeEx Spin Kit (Qiagen–USA, Valencia, CA) and resolved on a 377 ABI Automated DNA Cycle Sequencer (Applied Biosystems).

### Phylogenetic Analysis

Sequences were edited by using Sequencher Software v.3.1 (Gene Codes Corp., Madison, WI) and aligned with the Se-Al sequence alignment editor v.1.0 (http://evolve.zoo.ox.ac.uk/software.html?id=seal). Sequences from DRC collected in 1997 (*1*) were truncated to align with our C2V3C3 sequences, and gaps and ambiguous positions were removed, resulting in an alignment of 304 nt sequences; the first 60 nt represented the C2 region. For the outgroup in our tree, a consensus sequence representing the most common nucleotide at each position for each subtype represented in the Zaire/DRC dataset was selected, and then an overall DRC consensus sequence (DRCcons) was inferred. No positions were undefined in any of the consensus sequences, and no unclassified or unresolved (subtype assignment) sequences were included in the consensus sequence calculation. The Modeltest program, v.3.1 (*18*) was used to test for a statistically justified model of DNA substitution. The model of evolutionary change used in the tree was Transversion Model (TVM) + g (0.8575), where g is the shape parameter of the γ distribution (heterogeneity among sites), and TVM is the model of substitution whereby A↔G = C↔T and the other four rates are unique. To search for the best tree with such a large dataset (280 taxa) using the DRCcons sequence as the outgroup, several relatively fast tree-building algorithms were employed: Weighbor (http://www.t10.lanl.gov/billb/weighbor/index.html), neighbor-joining (PAUP), BioNJ (PAUP), and Fitch (PAUP). Trees derived from these programs were input to the PAUP* (*19*) tree scores program to compare the generated log likelihood scores. The neighbor-joining tree program was selected for our phylogenetic analysis since it had the best log likelihood score, although it was not substantially better (Shimodaira-Hasegawa test) than the BioNJ or Weighbor trees.

### Genetic Distance Analysis

Subtype and CRF designations were determined from the phylogenetic analysis, and sequences grouping in subtypes were input into the MEGA program version 2.1 (http://www.megasoftware.net) to calculate the means and standard errors of the intrasubtype/intraCRF diversity, using the Kimura 2P distance model.

## Results

All 3,988 serum samples were tested by EIA, and 209 seroreactive samples were further tested by Western blot: 140 (3.5%) were HIV-1 seropositive, and 69 (1.7%) had indeterminate Western blot patterns. Samples from infected persons who have not yet seroconverted can display indeterminate Western blot patterns. If we were to assume that all 69 persons with indeterminate Western blot results were seroconverting at the time of sample collection, then the upper limit for the frequency of HIV infections within these early samples was still low at 5.2%. No HIV-2 EIA-positive specimens were confirmed by HIV-2 Western blot analysis.

The serum samples had been stored at –20°C for almost 2 decades, a temperature at which RNases are still active. Because we were concerned that the quality of the RNA would be compromised, we attempted to amplify three relatively short gene regions: the p17 region of *gag* (474 bp) and two *env* fragments, C2V3C3 (400 bp) and gp41 (460 bp). All serum specimens could not be amplified, most likely because of the poor quality of the samples. A comparison of intrasubtype diversity was determined for our 50 C2V3C3 sequences and for 181 DRC C2V3C3 sequences from 1997 (1, GenBank, http://www.ncbi.nlm.nih.gov/) with known subtype assignments (Table 1). Unclassifiable sequences were omitted from this analysis. We found the intrasubtype variability was already high in the mid-1980s (9.6%–18.7%) yet was significantly greater for each 1997 *env* subtype with sufficient numbers for comparisons (A, C, D, E, F1, G). No significant change was found in the frequency of each subtype (Table 1).

**Table 1:** Comparison of intrasubtype/CRF^a^ genetic distances and subtype distribution

Subtype/CRF	Kinshasa: mid-1980s			DRC: 1997		
	n	Nucleotide distance (%)	% frequency of subtype	n	Nucleotide distance (%)	% frequency of subtype
A	22	15.1	44	84	20.8^b^	46
C	3	13.1	6	18	18.0^b^	10
D	10	12.1	20	23	18.7^b^	13
CRF01-AE	3	9.6	6	4	17.9^b^	2
G	5	15.6	10	15	20.1^b^	8
F1	3	10.0	6	7	14.8^b^	4
H	2	18.7	4	16	23.5	9
J	1	–	2	7	21.2	4
K	1	–	2	7	20.4	4

^a^CRF, circulating recombinant forms; DRC, Democratic Republic of Congo. ^b^Significant difference (distances significance test = *t* test, frequency of subtypes significance test = Fisher exact test).

**Figure 1:**
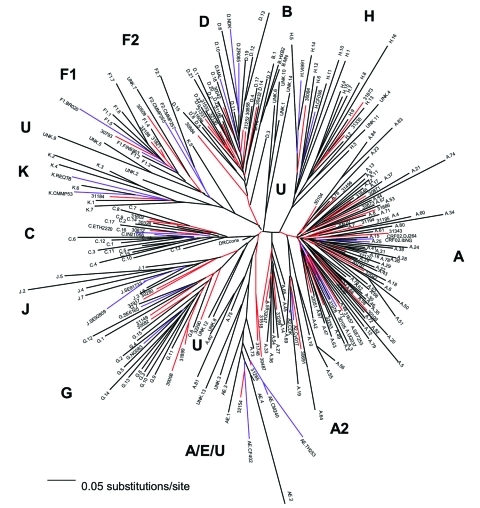
Neighbor-joining tree of 56 Zaire strains from the mid-1980s, 197 Democratic Republic of the Congo (DRC) strains from 1997, and subtype-specific reference strains. The number of nucleotides in the final alignment was 304 bp after gap stripping. The model of evolutionary change used in the tree was the Transversional Model (TVM) +g (0.8575), where g is the shape parameter of the γ distribution (heterogeneity among sites) and TVM is the model of substitution whereby A↔G = C↔T and the other four rates, all transversions, are unique. Our 1980s C2V3C3 sequences are indicated by a five-digit code, and their branches are red; the 1997 sequences have the subtype (as indicated in GenBank) as the first letter, followed by a one- or two-digit number or the designation unk = unclassifiable, and these branches are black; the reference strains have the subtype as the first letter followed by the reference name as follows: A.SE7253, A.92UG037, A2.CDK, A2.Cy017; B.HXB2, B.MN; C.ETH2220, C.IN21068; D.84ZR085, D.NDK, MAL; E.CD402, E.CM240, E.TH253; F1.FIN9363, F1.93BR020; F2.CMMP255, F2.CMMP257, G.NG083, G.SE6165; H.90CF056, H.VI991, J.SE91733, J.SE92809; K.96CMMP253, and K.97REQTB; these branches are purple. Subtype characterizations of the phylogenetic clusters in the tree are indicated in bold, lineages consisting of unclassifiable sequences are designated as U.

Figure 1 represents a neighbor-joining tree containing the following classifiable and unclassifiable C2V3C3 sequences: 56 of our early sequences from Kinshasa, 197 from the DRC collected in 1997 (subtype classifications taken from the V3–V5 sequences published in GenBank), and subtype-specific reference sequences. Some sequences that were difficult to classify in trees containing just our 1980s Kinshasa sequences and subtype-specific reference strains (data not shown) were easier to classify in the context of the larger combined Zaire/DRC phylogenetic tree. At least one of our early strains clustered with subtypes A, B/D, C, D, F (F1), G, H, J, and K, and the circulating recombinant form (CRF)01-AE clustered with 1997 CRF01 sequences. Also, some of our unclassifiable sequences clustered with 1997 unclassifiable DRC strains. One major clade contained subtype A sequences; however, at least four other distinct lineages branched independently in the tree, yet also contained 1997 sequences previously identified as subtype A viruses. One of these lineages that contained 1997 sequences previously designated as subtype A or unclassifiable also contained CRF01-AE strains in the apical portion of the cluster. Three additional unique lineages are present in the tree composed of unclassifiable sequences, perhaps representing new subtypes. Unclassifiable 1997 sequences also branched between the F1 and F2 lineages along with one of our 1980s strains, which suggests a continuum of diversity within subtype F strains instead of distinct sub-subtypes.

Even the node connecting the subtype B and D sequences was no longer distinct because of sequences falling basal to the two lineages, which made the subtype B strains appear to be part of a larger subtype D group. The 1997 sequence labeled D.3 in the tree [97DC.KS26 (accession no. AJ404096)] possibly contained a recombinant breakpoint, which caused it to branch very deep along the D lineage. However, our sequence 30884 also fell outside the node connecting subtypes B and D. In fact, this strain significantly clustered within the node connecting subtypes B and D in p17, C2V3C3, and gp41, yet did not significantly cluster with either subtype. A combined Zaire/DRC consensus sequence was constructed and used as an outgroup in this tree. The branch representing this sequence was very short and was located close to the center of the tree.

**Figure 2:**
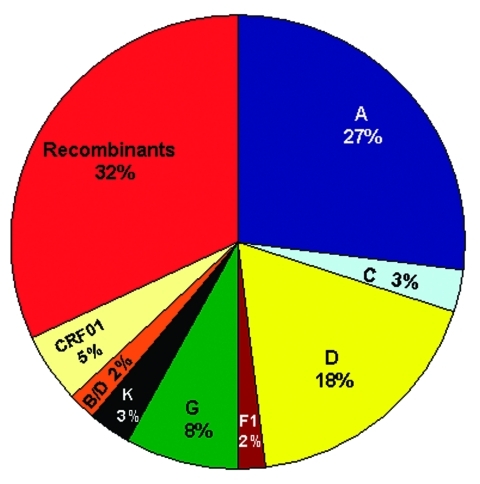
Distribution of subtypes and recombinant viruses. The pie chart represents 66 strains for which sequences from at least 2–3 gene regions were available for comparison; the subtypes in the pie chart represent concordant phylogenies suggestive of possible "pure" subtypes; the CRF01 and unique recombinant viruses are indicated in the pie chart. Table 2 summarizes subtypes of unique recombinant viruses.

To determine an estimate of potentially "pure" subtypes, we analyzed 66 of our mid-1980s HIV strains from which we were able to amplify and sequence two or three gene regions: 53 (80.3%) viruses were amplified in all three gene regions (p17, C2V3C3, and gp41). Figure 2 shows the viruses with concordant phylogenies (possibly pure subtypes) in the sequenced gene regions as follows: A (27%), C (3%), D (18%), F1 (2%), G (8%), K (3%), and our unusual B/D virus (2%). The remaining 37% of the strains represented recombinant viruses; 32% of these appeared to be unique recombinants, except where indicated in Table 2, and 5% were CRF01_AE, a mosaic lineage containing predominantly subtype A and a unique *env* lineage called E. Not only was this circulating recombinant form present by the mid-1980s, but only *env* subtypes A, D, and G were more prevalent. Some of the discrepant gene regions possibly indicate the presence of dual/multiple infections, and the sets of specific gene region primers selectively amplified different strains. However, since our hospital worker population does not represent a high-risk group, the prevalence of infections in Kinshasa was low, and recombinant viruses are generally rapidly observed following dual infections (*20*), we henceforth refer to samples with discordant gene regions as recombinant viruses.

**Table 2:** Subtypes of unique recombinant viruses shown in Figure 2.

Viruses^a^	Specific gene region		
	P17	C2V3C3	Gp41
n = 2	D	F1	F1
	G	A	A
	?	H	A
	A	H	G
	A	G	G
	A	A	G
n = 2	A	–	G
	?	–	G
	A	H	H
	A	A	?
	?	?	?
	C	C	?
	A	–	?
	F1	A	?
	A	?	A
n = 3	A	?	A
n = 2	J	?	?

^a^Except where noted, each row represents a single virus.

## Discussion

Despite a low frequency of HIV infections in Zaire in the mid-1980s compared to the level of infections currently observed in Africa, we found a remarkably high diversity of HIV-1 strains, with gene regions representing all the group M clades as well as unclassifiable regions. Intrasubtype diversity was already high among our samples, indicating that the HIV epidemic in Kinshasa was already mature by the mid-1980s. No significant change was seen in the frequency of group M subtypes between our early Kinshasa strains and a set of DRC samples collected in 1997 (*1*), despite political and social upheavals from 1986 to 1997.

We hypothesized that if the HIV epidemic originated in central Africa, phylogenetic analysis of our 1984/1986 Kinshasa and 1997 DRC C2V3C3 sequences might provide a more detailed history of HIV-1 evolution. We found a spectrum of diversity both within and between currently recognized HIV-1 group M subtypes and sub-subtypes (Figure 1). Some lineages, thought to represent discrete subtypes (or sub-subtypes) within the current global epidemic, no longer appeared distinct when analyzed in the context of the large diversity within Zaire/DRC sequences, i.e., multiple, distinct C2V3C3 subtype A lineages; new lineages containing unclassifiable strains; a continuum of diversity within and between the F1 and F2 sub-subtypes; and deep, unclassifiable branches. These findings demonstrate that the HIV diversity in the mid-1980s in Kinshasa was far more complex than in strains currently found in other parts of the world.

Another finding was the placement of C2V3C3 sequences of CRF01-AE in the apical branches of a lineage containing subtype A and unclassifiable sequences as basal branches. CRF01-AE is generally believed to be a recombinant virus with *gag* and *pol* genes sharing a common ancestry with subtype A, while the *env* and *vpu* genes are derived from a currently unknown parental strain, subtype E. However, others hypothesize that the unique subtype E portion of envelope is not a result of recombination (*21*) but may be because a higher evolutionary rate in the *env* and *vpu* genes make it unrecognizable as subtype A. Our data may be the first demonstration of subtype E *env* sequences grouping within a cluster of subtype A and previously unclassifiable sequences.

A combined Zaire/DRC consensus sequence used as an outgroup was very short and located near the center of the tree, close to the expected location of the hypothetical ancestor of the HIV-1 group M subtypes. This finding, along with the complexity of the combined phylogenetic tree, supports the theory that the group M subtypes evolved from a common ancestor, which may have originated in this part of Africa.

Since less than 1/10 of the genome was sequenced and analyzed in the present study, and recombination can occur anywhere along the genome, 37% recombinant viruses is very likely a significant underestimate of the actual frequency of chimeric strains cocirculating in Kinshasa by the mid-1980s. In fact, most HIV-1 strains may have contained recombinant genomes by that time. Unfortunately, our data do not allow us to discriminate whether this level of recombination through superinfection intensified the fitness of HIV strains at that time or merely contributed to viral diversity.

**Figure 3:**
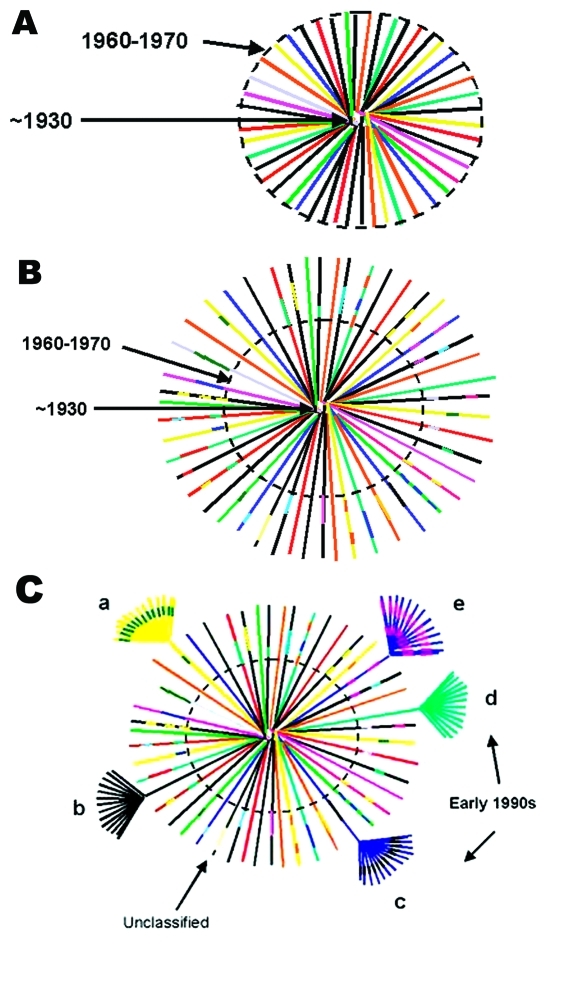
Hypothetical model of HIV-1, group M evolution. A. Star phylogeny representing the evolution of the ancestral HIV-1, group M virus that was able to adapt in humans and was transmitted among rural populations in central Africa from approximately the 1930s (*22*). Over time, the viruses would have become increasingly genetically distinct from each other and the original parental strain. The dotted circle denotes the beginning of migration from these remote areas to cities in central Africa (approximately 1960–1970). B. Recombinant lineages (outside the dotted circle), represented by multicolored lines indicating mosaic viruses or genetic mixes of the circulating strains, would have been the result of population migration, urbanization, patterns of sexual activity, and medical practices (two of the oldest, fully characterized sequences, MAL [1985] and Z321 [1976], were both recombinant viruses from Zaire). Recombinant viruses would have continued to be generated and transmitted until introduced into high-risk populations, such as commercial sex workers, taxi drivers, commercial truck drivers, or long-distance truck drivers, and then rapidly transmitted within and between these social networks. Such high-risk social networks throughout central Africa were responsible for the rapid expansion of a relatively small number of evolving viruses, including recombinant strains, locally, regionally, and eventually globally. These epidemiologic groupings are represented as clusters of highly related strains at the end of a few HIV-1 lineages. Panel C shows what phylogenetic analysis of global HIV-1 strains collected in the early 1990s, when sequence characterization first began, and after being exported out of central Africa, would have looked like. The clusters of related sequences from founder viruses, which were disseminated globally, would have appeared as subtypes or clades, arbitrarily labeled a–e. Occasionally, strains that were not widely expanded were identified and designated as unclassifiable. From this hypothetical modeling, and the high numbers of recombinant strains, it seems unlikely that only pure subtypes were exported from this region of Africa to establish mini-epidemics in other countries. Therefore, at least some of what we currently define as pure subtypes most likely arose from recombinant genomes originally generated somewhere in central Africa.

Although the precise ancestry of HIV-1 is still uncertain, it appears to have a zoonotic origin (*8**,**9*). Ecologic factors that would have allowed human exposure to a natural nonhuman primate host carrying a precursor virus to HIV were therefore instrumental in introducing the virus into humans. This fact suggests that zoonotic introductions of HIV may have occurred in isolated populations but went unnoticed as long as the recipients remained isolated (*11*) (Figure 3A). However, with increasing movements from rural areas to cities, which occurred in sub-Saharan Africa during the 1960s and 1970s, such isolation became, or was becoming, increasingly rare. The number of sub-Saharan African cities with >500,000 inhabitants rapidly increased from 3 in 1960 to 28 by 1980 (*23*). From 1965 to 1985, the proportion of the total population living in urban areas of central Africa rose from 21% to 35% and in western Africa from 17% to 29%.

Transmission of HIV would have amplified among high-risk populations, such as commercial sex workers, where HIV superinfections generating recombinant viruses could have resulted (Figure 3B). These recombinants could then have been spread to other, lower risk persons, such as men who visit female sex workers, their spouses, other heterosexual partners, and persons at highest risk for sexually transmitted diseases (*24*). Other transmission routes could also have helped create and spread recombinant viruses. For example, in 1986, studies from Kinshasa showed a strong association between receiving medical injections and HIV seropositivity among HIV-seropositive infants born to seronegative mothers (*25*), among hospitalized children 2–14 years of age (*26*), and among healthcare workers (*27*). Demand for blood transfusions was also high because of malaria-associated anemia, pregnancy-related complications, and sickle-cell anemia (*28**,**29*); at this time, blood was generally not screened for HIV infection (*30*). Therefore, many factors associated with population migration and urbanization, patterns of sexual activity, and medical practices could have played roles in producing and spreading high numbers of recombinant viruses in Kinshasa by the mid-1980s.

When HIV subtypes were initially genetically characterized in the early 1990s, the first identified viruses were assumed to represent pure subtypes, and viruses found afterward were compared to these prototypic strains. However, our data demonstrate that substantial intersubtype recombination had already occurred at the time when HIV-1 viruses were initially classified (Figure 3C). With so many recombinant viruses present at the cusp of the global HIV epidemic, at least some of the recombinant viruses in central Africa were likely classified as pure subtypes after being exported from Africa and establishing regional epidemics in other parts of the world. This realization could affect our current understanding of the range of diversity within the HIV epidemic (http://hiv-web.lanl.gov/content/hiv-db/REVIEWS/PEETERS2000/Peeters.html); of immunogen selection for vaccine design (*31**,**32*), especially in light of superinfections involving multiple subtypes (*33*); and of mathematical models based on molecular clocks and nonrecombinant strains (*22**,**34**,**35*) for dating the introduction of HIV into humans.
